# ‘Pastoral practices’ for quality improvement in a Kenyan clinical network

**DOI:** 10.1016/j.socscimed.2017.11.031

**Published:** 2017-12

**Authors:** Gerry McGivern, Jacinta Nzinga, Mike English

**Affiliations:** a Warwick Business School, University of Warwick, Coventry CV47AL, UK; b KEMRI Wellcome Trust, Nairobi, Kenya; c Nuffield Department of Medicine, University of Oxford, UK

**Keywords:** Kenya, Clinical networks, Leadership, Clinical governance, Quality improvement, Governmentality, Pastoral power, Low and middle income countries

## Abstract

We explain social and organisational processes influencing health professionals in a Kenyan clinical network to implement a form of quality improvement (QI) into clinical practice, using the concept of ‘pastoral practices’. Our qualitative empirical case study, conducted in 2015–16, shows the way practices constructing and linking local evidence-based guidelines and data collection processes provided a foundation for QI. Participation in these constructive practices gave network leaders pastoral status to then inscribe use of evidence and data into routine care, through championing, demonstrating, supporting and mentoring, with the support of a constellation of local champions. By arranging network meetings, in which the professional community discussed evidence, data, QI and professionalism, network leaders also facilitated the reconstruction of network members' collective professional identity. This consequently strengthened top-down and lateral accountability and inspection practices, disciplining evidence and audit-based QI in local hospitals. By explaining pastoral practices in this way and setting, we contribute to theory about governmentality in health care and extend Foucauldian analysis of QI, clinical networks and governance into low and middle income health care contexts.

## Highlights

• Pastoral practices influence health professionals to implement quality improvement.
• Local evidence-based guidelines and audit processes provide a foundation for QI.
• A constellation of network leaders and local champions inscribed QI into practice.
• Discussion in network meetings facilitated reconstruction of professional identity.
• Professionals disciplined their own use of evidence and audit for QI purposes.

## 1 Introduction

There has been excitement about the potential of quality improvement (QI) for enhancing global health and calls for a ‘quality revolution’ in health care (Kruk et al., 2016). Yet, despite the existence of QI methodologies and some understanding of QI barriers and facilitators (Batalden and Davidoff, 2007, Buckley and Pittluck, 2015), we know little about how to develop social and organizational processes to convince professionals to implement QI into practice (Berwick, 2012, Hanefeld et al., 2017).

Evidence-based medicine, based on a dominant positivist epistemology, has become ‘the gold standard’ in health care. Yet implementing evidence into clinical practice is often slow and contested, complicated by professional power, politics, social norms and contextual conditions (Greenhalgh et al., 2004; S. Timmermans and Berg, 2003). By explicitly considering the role of power in the social construction of knowledge, Foucauldian theory may explain how evidence and QI are produced and why they may, or may not, be implemented.

One QI strategy is the development of clinical networks (Flynn, 2002), providing lateral and relational forms of governance. Clinical networks connect professions and organisations, aiming to diffuse evidence, best practice, expertise and learning across health systems, and thus facilitate standardised high quality care. However, clinical networks rely on good leadership and network leaders often have no hierarchical authority, so their leadership must *influence* improvement by changing how network participants understand themselves and what they do (Addicott et al., 2006, Provan and Milward, 1995).

Recent research (Ferlie et al., 2013, Waring and Martin, 2017) suggests that clinical network leaders may influence change by exercising what Foucault (2007) describes as ‘pastoral power’ and constructing a shared ‘governmentality’. This provides a novel way of conceptualising the organisational and social processes facilitating QI. However, this nascent explanation requires theoretical development and testing in different empirical contexts. Moreover, little research has examined clinical networks using Foucauldian analytical frames in low and middle income countries (LMICs) (Ferguson and Gupta, 2002, Lemke, 2011), where networks may provide an effective mode of clinical governance in the absence of governments able to change or regulate behaviour (De Herdt and Oliver de Sardan, 2015).

We use the Foucauldian concept of ‘pastoral practices’ (Waring and Martin, 2017) to explain the construction of governmentality and related QI processes within a Kenyan paediatric clinical network. Below, we discuss Foucauldian theory about governmentality, pastoral power, pastoral practices and how these have been used to explain clinical networks and QI. We then describe the network we studied, our qualitative research methods, and empirical findings. Finally, we highlight our contribution and its implications for theory, policy and practice.

## 2 Governmentality, pastoral power and their application in health care

Foucault's early work examined the interrelationship between power and knowledge, using medicine as a prime example. Foucault explained how taken-for-granted truths, which both enable and constrain thought and action, were constructed by institutionalised modes of categorising, ordering and ranking, which emerged from historical struggles between actors promoting competing truths (Elden, 2017, Foucault, 2008). Thus, Foucault argued that scientific method for ‘discovery of truth is in reality a certain modality of the production of truth’ (Elden, 2017: 185). Foucault (1977) then described how by making individuals knowable and visible within organisations (using ‘panopticon’ prisons and hospitals as examples), ‘disciplinary power’ led individuals to internalise and regulate their own behaviour according to institutionalised categories, modes of ordering and social norms.

While Foucault's (Foucault, 1977, Foucault, 2008) ideas inspired research exposing ‘technologies of domination’, he was clear about the need to ‘cease’ describing the effects of power in negative terms (e.g. excluding, repressing, censoring and concealing), noting that ‘power produces’ knowledge, individuals, reality and truth in ways that may also benefit individuals and society (Foucault, 1977: 194). Indeed, Foucault's (1993) final ideas explored ‘techniques of self’ permitting individuals to cultivate their own identities.

Foucault (2007) developed the concept of ‘governmentality’, which linked technologies of self and technologies of domination, to explain transition from sovereign states, ruled by force, to neo-liberal states, governed at a distance through ‘practices of freedom’ (Rose, 1999). Foucault (2007: 108) defined governmentality as ‘the ensemble formed by institutions, procedures, analyses and reflections, calculations and tactics … that has the population as its target, political economy as its major form of knowledge and apparatuses of security as its essential technical element’.

In simpler terms, governmentality explains mechanisms through which governments impose their will on citizens, who internalise the ‘mentality’ of ‘government’, come to think of themselves as part of a population, and regulate their behaviour in the collective interest. Governmentality explains how, by inciting, inducing, seducing, and making various actions easier or harder, governments are able to allow citizens to make the ‘right decision’, negating the need for direct external control (Dean, 1999, Lemke, 2011, Rose, 1999).

Dean (1999) distinguishes four ‘dimensions of governmentality’ relating to ways of: (1) seeing, perceiving and making things visible; (2) thinking, questioning and producing truth forming ‘the episteme of government’; (3) acting, intervening and directing, practical rationalities, modes of expertise, mechanisms, techniques and technologies; and (4) ways of forming subjects, affecting individual and collective identities. He notes: ‘regimes of government … elicit, promote, facilitate, foster, and attribute various capacities, qualities and statuses to particular agents. They are successful to the extent that these agents come to experience themselves through such capacities’. (Dean, 1999: 32).

Foucault's (2007) related concept of ‘pastoral power’ explains the processes through which governmentality is internalised. Pastoral power draws on the metaphor of the relationship between pastors and their congregation, with pastors (‘shepherds’) acting as intermediaries between Christian discourse and the Christian community (their ‘flock’). Pastors are accountable for inculcating moral behaviour, so the behaviour of their community determines the reputation of the pastor, who achieves their own salvation through the salvation of the flock.

Pastoral power also explains the relationship between discourse, individual subjectivities and behaviours in other settings. For Dean (1999), pastoral power can be thought of as about cultivating ethical behaviours benefitting collective social welfare. Thus, contemporary pastors may include experts or therapists promoting socially or clinically desirable behaviour (Rose, 1999). Indeed, Foucault (2007: 199) notes: ‘In its modern forms, the pastorate is deployed to great extent through medical knowledge, institutions and practices’.

In health care, quality regimes (Flynn, 2002, van Rensburg et al., 2016), patient safety initiatives (Martin et al., 2013, Waring, 2007), evidence-based medicine (Ferlie and McGivern, 2014, Ferlie et al., 2012) and transnational diffusion of evidence, research and practices (Ferguson and Gupta, 2002, Geissler, 2015) have been explain as forms of governmentality. Clinical networks have also specifically been explained in terms of governmentality (Ferlie et al., 2012, Ferlie et al., 2013, Flynn, 2002, Waring and Martin, 2017).

Drawing on Dean's (1999) four dimensions, Ferlie et al. (2013) argue that effective clinical networks operate through evidence-based governmentality, involving the assemblage of four elements: an *episteme* framed in relation to evidence-based guidelines; practices and mechanisms linked to clinical audit making health care provision and outcomes *visible*; local *technical* processes through which guidelines and clinical audit are enacted; and their use to shape individual and collective professional *identities* in a way facilitating reconfiguration and improvement of health care services. Relatedly, Ferlie and McGivern (2014) explain network leaders exercising pastoral power, using clinical audit to make performance visible, thus disciplining doctors to use evidence-based standards to maintain their professional identity.

Developing the application of governmentality and pastoral power in health care further, Waring and Martin (2017) describe four ‘pastoral practices’ shaping identities and behaviours in clinical networks: (1) ‘*Constructive practices*’, identifying and re-coding rationalities, translating the ‘scripture’ of evidence in a way relevant and comprehensible to local communities; (2) ‘*Inscription practices*’, involving ‘sermon’ like communication and framing, encouraging network members to internalise re-coded discourses; (3) ‘*Collective practices*, whereby ‘pastors’ shape and frame subjectivities for the wider ‘flock’, defining and reinforcing collective boundaries. This encourages communities to collectively control behaviours, extending Foucault's concept of ‘technologies of the self’ to ‘technologies of the collective’. Accordingly, professionals develop their collective social identity through socialising as a professional community; and (4) ‘*inspection practices*’ in which ‘pastors’ provide ongoing guidance to the community, identifying practices and subjectivities conforming with or deviating from acceptable behaviours, and in doing so creating, maintaining or disrupting social order.

Governmentality scholars have been criticised for ‘Eurocentrism’ (Ferguson and Gupta, 2002, Lemke, 2011). Similarly, while there is some research on governmentality in health care in LMICs (Brown, 2016, Geissler, 2015, van Rensburg et al., 2016), we know little about its role in clinical networks and QI in LMIC health care contexts. Thus, we analyse governmentality in a Kenyan clinical network using theory about pastoral practices.

## 3 The Clinical Information Network

The ‘Clinical Information Network’ (CIN) is a paediatric health care network spanning 14 Kenyan public district hospitals, aiming to improve health care for Kenyan children. CIN operates within the Kenyan Medical Research Institute (KEMRI) – Wellcome Trust Research Programme (KWTRP). The network developed from collaboration between researchers, the Kenyan Ministry of Health and the Kenyan Paediatric Association, focused on adoption of recommended evidence-based practice and overcoming barriers to their adoption locally and collectively. CIN held its first formal network meeting in 2013 (English, 2013, English et al., 2011, English et al., 2017).

As in many LMICs (Chandler et al., 2009, Willis-Shattuck et al., 2008), the quality of health care, morale and motivation of clinical staff in Kenya are often low (English, 2013, English et al., 2011). Common illnesses (e.g. diarrhea, pneumonia, malnutrition and malaria), account for most deaths among Kenyan children, even though they can be prevented and treated using evidence-based guidance about diagnosis and treatment (World-Health-Organization, 2016). Accordingly, CIN promotes and supports development and use of evidence-based guidelines, measurement of clinical activities and outcomes, leadership and a form of QI involving meeting and discussing the practical challenges of delivering QI and locally solving problems highlighted by audit.

## 4 Methods

This paper is written by an interdisciplinary team containing CIN insiders and outsiders. A social scientist from outside CIN, funded by the network to research and explain how it functioned, and a social scientist based at KWTRP, collected and analysed data. A clinician involved in CIN, who commissioned the research, helped interpret findings but was not involved in data collection or analysis. Accordingly, rather than providing an objective external evaluation of CIN, the research aimed to develop a formative explanation and theory of change for the organisational processes underlying network activities, developed through interdisciplinary, insider-outsider dialogue.

We received ethical approval for our empirical research from the KEMRI Scientific and Ethical Review Unit in Kenya. We then conducted a qualitative case study of the CIN, examining its creation (in 2013) and functioning at the time of data collection (2015–16), drawing on interviews, ethnographic observation and documentary analysis.

We conducted semi-structured interviews with 34 health professionals involved with CIN, half individually and half in groups. Groups contained members of a single profession at the same grade to minimise the potential of inter-professional or hierarchical relations inhibiting open speech. We asked questions about interviewees' experiences of Kenyan health care generally and CIN specifically, including the network's impact on health professionals, patients and the hospitals involved. Interviews lasted 22–90 min, were audio-recorded and transcribed.

Interviewees included: two CIN leaders (interviewed individually); 12 consultant paediatricians (10 interviewed individually; two together); nine nurses ‘in-charge’ of paediatric departments (interviewed in three groups); a medical officer (junior doctor - interviewed individually); seven Health Records Information Officers (HRIOs, interviewed in two groups of three and one individually); a medical epidemiologist and representatives of the Kenyan Paediatric Association and the Kenyan Ministry of Health (all interviewed individually).

We observed three bi-annual CIN meetings, including how network leaders engaged with participants, training and discussion of evidence-based guidelines and data collection, and participants' reactions. Informal conversations with participants also informed our understanding of CIN. We conducted some formal interviews before and after CIN meetings. We analysed a detailed description of CIN's activities (English et al., 2017) and other CIN documentation.

We took an abductive approach (S. Timmermans and Tavory, 2012) to data analysis and theorisation. Interview questions were framed by research on clinical networks in high income countries, while exploring CIN activities and their impact. We analysed and coded data looking for patterns of responses (Strauss and Corbin, 1998). We then went through an iterative cycle comparing theory and data, looking for fit and anomalies, to develop a theoretical model explaining our case.

We initially noted similarities between our data and theory explaining clinical networks using governmentality theory (Ferlie et al., 2013), which focused our attention on Foucauldian health care literature. Reviewing this literature, we found Waring and Martin's (2017) model of ‘pastoral practices’ provided a theoretical frame potentially explaining our data, which we refined to better explain our case, highlighting new aspects of pastoral practices and extending their generalisability to LMICs. Finally, we developed Table 1 to illustrate empirical data, theoretical codes and concepts, which also helped us interpret our findings and construct our theoretical model (Gioia et al., 2012).

**Table 1:** Pastoral practices within CIN.

Pastoral practices	Pastoral practices within the Network		Illustrative narratives
**Constructive practices**	**Constructing evidence-based episteme;** Adapting & adopting international evidence & disseminating local guidelines		*“WHO came up with the guidelines I was … helping in adopting and adapting the guidelines to the Kenyan needs.’* (Network Director) *“We had started to adopt more rigorous approaches to the evidence collection … reviews … discussing … draft recommendations with the … paediatric community … [then] disseminating these guidelines.”* (Network Director)
	**Constructing practices making health care provision & outcomes visible;** Establishing local audit & data collection		*“The training involved [district hospitals] doing a self-assessment … We would go back with the survey results … discuss those … to get them to both acknowledge the problems … [and] come up with action plans.”* (Network Director)
	**Establishing pastoral status** by constructing a local episteme		*“We put together national guidelines … that process … introduced me to … key stakeholders … what we had been up to was more widely known because of … disseminating these guidelines.”* (Network Director)
**Inscription practices**	**Championing**	Change	“*Challenging mind-sets … becoming a champion, going out speaking, teaching.”* (Network Director)
		Evidence-based episteme	*“I tell [medical interns] that the protocol is our paediatric bible.”* (Paediatrician)
		Visibility practices	*“There was very poor documentation … data is useless if the diagnosis of the patient is entered wrong and … posted in the Ministry of Health … it doesn't inform anything, it actually confuses allocation of resources.”* (Network Director) *“Reach the people who are in charge of paediatric care. Through improving their knowledge and monitoring their performance, then I believe you can have a greater impact.”* (Epidemiologist)
	**Demonstrating**	Change	*“Role model and reinforce … change the ‘norm’ … creating a critical mass.”* (Network Director)
		Evidence-based episteme	*“We've been forced to actually take [interns] through it and even as we do our wards rounds we ask them questions from those guidelines.”* (Paediatrician) *“There's a perception that if you're … checking guidelines you're not good enough … now [interns] realise that even the consultant refers to it, then it's not a weakness, so that mind-set change.”* (Paediatrican)
		Visibility practices	*“[Network directors] keep on giving a feedback … come in physically [to district hospital] … it is very useful.”* (Nurse-in-charge)
	**Supporting & mentoring**	By network leaders	*“Promote and encourage rather than say you have got to do it this way … put yourself in the shoes of the person you're trying to support or influence … appreciate their realities … emphasize the positive.”* (Network Director) *“Support is very important … to guide and giving the feedback … help improve data … quality of care.”* (Nurse-in-Charge) *“[Network directors'] mentoring, that supervision continues and it is helpful.”* (Paediatrician)
		By wider constellation of leaders	“*I mentor a lot of doctors … I love paediatrics … I'm passionate about what I do, including even quality improvement … it's kind of catching.”* (Paediatrican)
**Collective practices**	**Meeting & sharing as a professional community**		*“Being able to engage with one's peers … having meetings … for the profession to begin to take … a view … a stand on … own that agenda.”* (Network Director) *“CIN has been great. It has made me meet so many other people from different organizations and different work backgrounds but we are all pushing towards the same goal.”* (Nurse-in-charge) *“Coming together and share challenges … successes … learn … we move together to improve the quality of care for our children [patients], individually and then collectively.”* (Paediatrician)
	**Collectively championing & demonstrating evidence-based professionalism;** collectively developing a professional identity		*“[CIN] fitted into that generational issue, of the expanded internet … seeing that there is more than just doing what you were told fifteen years ago.”* (Network Director) *“Consultants' … traditional way of teaching … [is] to intimidate everybody … to consult them you need to think twice … [Now] we can generate our own evidence … [do] critical appraisal.”* (Paediatrician)
**Inspection practices**	**Disciplining**	Evidence-based episteme	*“Somebody watching how do you do things, you become better and conscious*.” (Nurse-in-charge) *“Keep checking [interns] in the rounds then they know that it is checked. Unfortunately, that is what it takes to get some people to use guidelines.”* (Paediatrican) *“Medical Officers interns … are not listening to experienced nurses … you are able to discipline them and put them straight when they are doing wrong things … [giving] out the standards on the wards as expectations.”* (Nurse-in-charge)
		Audit & data collection	*“CIN is like someone coming to audit me … my role has been to rectify that which has been pointed out by CIN as a problem in the hospital. So, for me it is a fantastic thing.”* (Paediatrican) *“It is kind of competition when you get the feedback [on] … how you are performing, look at the other hospital, you get that feeling we should also be there [performing better].”* (Nurse-in-Charge)

## 5 Empirical case study: pastoral practices within CIN

We present our empirical findings, structured around four pastoral practices below.

### 5.1 Constructive practices: constructing an evidence-based episteme, mechanisms and practices making health care provision and outcomes visible, and pastoral status

A first dimension of governmentality is the *episteme -* system of knowledge or way of thinking (Dean, 1999) – that clinical networks activities are based on (Ferlie et al., 2013). CIN's leaders, both consultant paediatricians and medical academics, had been involved in developing Kenyan evidence-based paediatric and neo-natal guidelines at national level, adapting WHO guidelines to their local context. CIN director 25 noted: *“We had started to adopt more rigorous approaches to evidence collection … reviews … discussing … draft recommendations with the … pediatric community … [then] disseminating these guidelines.”* Thus, CIN leaders had engaged in *constructive pastoral practices* to develop a local evidence-based episteme.

A second dimension of governmentality is *visibility* (Dean, 1999), also found to be important in constructing governmentality in clinical networks (Ferlie et al., 2013). While clinical audit, data collection and performance measurement are pervasive in high income country health systems, in Kenya, and many other LMICs, there is little reliable data about health services' provision or outcomes (Kihuba et al., 2014, Tuti et al., 2016). Moreover, data collected is rarely used by clinicians to improve care or by health care managers to hold clinicians to account in LMICs (English, 2013). As a Paediatrician (2) noted: *“The Ministry [of Health] actually collect a lot of data … but using it has been a challenge and we don't even get feedback.”*

To remedy this problem, CIN engaged in constructive practices, developing clinical audit and data collection mechanisms, tools and practices making visible how Kenyan children were diagnosed and treated, and the consequent clinical outcomes achieved. As part of this process, CIN developed and implemented tools capturing trustworthy data for quality monitoring against relevant and agreed indicators, a standardized admission record, changed record systems in district hospitals involved, and devised systems to feedback results. CIN encouraged clinicians to collect more data about patients' generic health status, for example, children's HIV status and screening for malnutrition (English et al., 2017). Every two to three months CIN then provided written feedback to network hospitals on their performance. Thus, it was intended that ‘harnessing data as a disciplinary force’ (English, 2013: 10) would support QI.

Developing Kenyan national guidelines involved extensive engagement with key stakeholders, including the Kenyan Ministry of Health, Kenyan Medical Schools and the Kenyan Paediatric Association. CIN Director 25 noted: *“That process of developing the national guide … introduced me to … stakeholders … that needed to be engaged … [who] began to know me and what I was up to.”* Thus, involvement in constructive practices indirectly established CIN leaders' expert and legitimate *pastoral status*. Yet this pastoral status was also grounded in experience of the challenges Kenyan clinicians faced. Both CIN leaders had practised in the Kenyan health systems for over 20 years and taught Paediatrics and Child Health at a leading Kenyan university, so knew many Kenyan paediatricians personally. Thus, CIN leaders had expert, professional and personal credibility.

CIN members described both CIN leaders in inspirational terms, who, like pastors, inspired others to get involved in their mission. Consequently, other paediatricians joined a national guidance review panel or began locally evangelizing the practices that CIN promoted, fulfilling one of CIN's key aims to create leaders serving as evidence and audit-based champions in their local settings (English et al., 2017).

### 5.2 Inscription practices: championing, demonstrating, supporting and mentoring

Simply making clinical guidelines available does not ensure they are used (Nzinga et al., 2009). Indeed, having developed Kenyan paediatric guidelines, CIN Director 25 conducted research on whether clinicians in Kenyan hospitals were practising in-line with guidelines, which “*suggested major challenges”.* Thus, CIN Director 25 faced a dilemma: *“Either we start developing work to address some of those challenges, or … go back into a … technical bubble … develop the right guidelines but not worry too much about whether anybody ever use them. So that's when I became more interested in the implementation side of things.”*

From data analysis, we noted three pastoral inscription practices in CIN inscribing paediatric guidelines into routine care. The first involved CIN's leaders evangelically *championing* CIN's purpose as benefitting network participants and patients. CIN Director 1 described: “*Challenging mind-sets … becoming a champion, going out speaking.”* CIN Director 25 was described as *“the force behind what we are doing”* and *“our champion”* (CIN Director 1). Thus, by championing CIN's activities, its leaders motivated doctors and nurses involved in the network to use guidelines, clinical audit and data collection for QI purposes.

We noted a wider range of health professionals also involved in championing. All CIN participants we interviewed agreed that using evidence-based guidelines was an important means for improving the quality of health care. A few interviewees likened the paediatric guidelines to a ‘bible’. For example, Paediatrician 13 commented: *“I tell [medical interns] that the protocol is our pediatric bible and everyone has it in their pockets”*. This was helpful for training interns: *“The guidelines have been very useful for teaching the younger colleagues … interns coming into the department, that is the first thing we actually give them”* (Paediatrician 11).

CIN leaders also championed clinical audit, data collection and correctly documenting results: *“Data is useless if the diagnosis of the patient is entered wrong and … posted in the Ministry of Health … it doesn't inform anything; it actually confuses even allocation of resources”* (CIN Director 25). Again, they were supported by a range of professional champions within CIN. For example, Epidemiologist 23 noted: *“If you can reach the people … in charge of pediatric care, through improving their knowledge and monitoring their performance, then I believe that … you can have a greater impact on the outcome of children.”*

Reflecting research on clinical networks in high income health settings (Addicott et al., 2006, Provan and Milward, 1995), championing in CIN involved ‘influencing’ rather than imposing change. CIN Director 25 noted: *“We … promote and encourage rather than say you have got to do it this way … put yourself in the shoes of the person you're trying to support or influence, so that you can appreciate their realities.”* Similarly, Epidemiologist 23 noted*: “within CIN, where you have paediatricians who are authorities in their field, the type of leadership that they need is one of influencing, not teaching or directing what they should do … you can achieve significant changes in care through influencing” (Epidemiologist 23).*

A second pastoral inscription practice, again involving a wider constellation of health professionals, was *demonstrating,* in the sense of both teaching and role modelling change. While CIN provided training on clinical guidelines, data collection and QI techniques, their implementation required paediatricians to demonstrate their use in routine hospital care locally. Paediatrician 11 noted: *“We've been forced to actually take [interns] through [guidelines] … as we do our ward rounds, we ask them questions from those guidelines.”* Another commented: *“[Consultants] refer [to guidelines] a lot in the first month then [interns] just internalize them, so it becomes very easy to manage the conditions”* (Paediatrician 12). Paediatricians role modelled use of guidelines too, as Paediatrician 2 noted: *“There's a perception that if you're … checking guidelines you're not good enough … now [interns] realise that even the consultant refers to it, then it's not a weakness, so that's mind-set change.”*

Normalizing data collection and clinical audit involved pastoral inscription practices too. CIN provided on-going training for data clerks, HRIOs and clinical teams about how to collect data and understand CIN quality care reports (English et al., 2017). CIN leaders provided performance feedback to participating hospitals in person for the first 18 months, to ensure that participants could interpret data and use it to address problems, improve practices and clinical outcomes (English, 2013, English et al., 2011, English et al., 2017). Nurse-in-charge 30 commented that CIN's leaders: *“keep on giving feedback … they come in [to district hospital] physically … to instill more confidence and more value to the program. I think it is very useful.”* Accordingly, through demonstrating and role modelling, data collection came to be understood as an important component of QI.

A third inscription practice was *mentoring and supporting use* of audit for QI as a form of professional development (English et al., 2017). Nurse-in-Charge 5 commented: *“Support is very important … people to guide and giving the feedback … to help improve data … quality of care … mentoring … I find it very helpful.”* Likewise, Paediatrician 4 noted: *“[CIN leaders] will give you an answer that actually opens up a whole road of possibilities … they're really good mentors.”* Mentoring and support was also apparent during CIN meetings: *“The mentoring and supervision is useful, especially from the network coordinator … you see a lot of engagement … during [the CIN] forum … between the members of the network, the clinicians and their mentors.” (Epidemiologist 23).*

Moreover, senior paediatricians and nurses ‘in-charge’ locally mentored colleagues to adopt the practices CIN advocated. Paediatrician 4 commented: “*I mentor a lot of doctors … I love paediatrics … I'm passionate about what I do, including even quality improvement … it's kind of catching.”* Consequently, QI practices spread via a constellation of CIN members through the inscription practices we describe.

### 5.3 Collective practices: meeting and sharing as a professional community, championing and demonstrating an evidence-based collective professional identity

In Kenya, health professionals often work in remote district hospitals, overseeing clinical departments with little support or training. Many find this difficult, particularly without resources to provide high standards of care. Consequently, loss of motivation and burnout are common (Brown, 2016, Mbindyo et al., 2009). CIN leaders attempted to create a supportive network community to addressed this problem, by providing physical and online spaces in which to *meet and share as a professional community*. CIN encourages network participants to think about new approaches to problem-solving, and then implement, test and measure solutions, and facilitates sharing of learning across participating organizations, thus functioning as an improvement collaborative (English, 2013).

Bi-annual network meetings helped to develop paediatrics as a professional community in Kenya. CIN Director 25 described one of the network's aims as: *“Truly being able to engage with one's peers … for the profession to begin to take more of a view … a stand on what is happening and begin to own that agenda.”* CIN meetings enabled participants to share experiences, learn from colleagues facing similar challenges and develop as part of a multi-professional and multi-organisational paediatric community. Nurse-in-charge 30 noted: *“CIN has been great. It has made me meet so many other people from different organizations and different work backgrounds but we are all pushing towards the same goal.*” Similarly, Paediatrician 10 described how by “*coming together”* and being able to *“share challenges and successes and learn from others”* CIN members *“move together to improve the quality of care for our children [patients], individually and then collectively.”*

CIN meetings also facilitated the *championing and demonstrating of a new evidence-based collective professional identity*. English et al. (2017: 13) note: ‘an overarching theme of the network is to change the social milieu in which clinical leaders in hospitals operate. A broad professional focus on adoption of guidelines and improvement in care that is endorsed by recognized institutions and professional associations and an effort to create ownership of this agenda are at the heart of the network strategy.’

CIN leaders attempted to link recognition of senior colleagues and peers to affirming professional values, challenging a traditional, authoritarian medical culture in Kenya, which was seen to perpetuate outdated clinical practice. CIN Director 25 noted that CIN *“benefitted from trying to present … a new way of doing business”,* which junior health professionals were *“receptive to” due to a “dissatisfaction with the sort of old professor stands in the corner and tells you … [CIN] fitted into that generational issue, of the expanded internet … seeing that there is more than just doing what you were told fifteen years ago.”* This new approach appealed to younger health professionals.

Paediatrician 11 commented that traditionally Kenyan consultants “intimidated” medical trainees to the point that they never questioned what consultants told them. However, after involvement in CIN, junior doctors now: *“appreciate there is something called evidence, and evidence doesn't need to come out from out there, we can generate our own evidence … [and] critically appraise [evidence].”*

By meeting as a professional community, CIN members internalised governmentality associated with the evidence-based episteme, visibility mechanisms and practices, regulating their individual and collective clinical practices accordingly.

### 5.4 Inspection practices: disciplining evidence-based care and data collection

Finally, pastoral practices within CIN involved senior doctors and nurses using *inspection practices* to discipline junior medical interns to use guidelines. As a paediatrician (2) noted: *“If you keep checking them [interns] in the [ward] rounds then they know that it is checked. Unfortunately, that is what it takes to get some people to use guidelines … people are not used to checking guidelines.”* Nurse-in-charge 5 used guidelines to *“discipline … medical officer interns … not listening to experienced nurses … put them straight when they are doing wrong things”,* giving out *“standards on the wards as expectations from them.”*

Many CIN participants drew upon the visibility mechanisms of audit and data collection and related pastoral inspection practices to discipline themselves. Paediatrician 27 noted: *“It has changed the way I practise … we were audited and we discovered we are actually not doing a hundred percent … previously we were not paying attention to detail but now we are.”* Nurse-in-charge 14 commented: *“Someone coming to check on what you are doing … When you have a trigger of somewhere somebody watching how do you do things, you become better and more conscious*.” Paediatrician 13 noted: *“CIN is like someone coming to audit me … an external supervisor … coming to see how things are being done … to make you rectify the wrong things … for me it is a fantastic thing.”*

Interviewees also commented that provision of data, with hospital performance “*always compared with the rest”* (medical officer, 29), engendered a sense of competition between hospitals participating in CIN: *“It is a kind of competition when you get the feedback and look at graphs how you are performing, look at the other hospital”* (Nurse-in-Charge 5).

Moreover, unlike in the wider Kenyan health system, there appeared to be a sense of ownership of data collection within CIN because: *“It's very clear, [CIN] it's actually trying to use data to improve the quality of care … we own it … the fact that I don't have to send data to KEMRI does not stop me from collecting my own data”* (Paediatrician 4). HRIO 29 commented: *“I'm very passionate about CIN because, as our vision says … we are using this data, which we are generating to improve the quality of services to patients.”* Nurses were also described as *“owning”* CIN data and using it to *“assess the nursing work … holding each other accountable”* (Paediatrician 11).

However, we did note resistance among nurses to collecting data for CIN. Paediatrician 12 noted: *“Some [nurses] … tick information and … I feel like probably they didn't do it.”* Nurses explained that they were often too busy focusing on clinical care to document data: *“The workload is just too much … [Nurses] don't have time … observations are not well documented … [nurses] concentrate on giving treatment or attending emergencies”* (Nurse-in-Charge 14).

Some CIN members believed such resistance required further pastoral intervention. For example, HRIO 26 noted that *CIN's “biggest challenge”* was clinical staff who *“don't understand the purpose or the benefits of data” and* so provided *“data which is inaccurate”* or *“incomplete*”. The HRIO added that these clinical staff needed to understand that this was not *“good for decision-making and that's why the country is not moving ahead.”* Again, here we see data collection constructed in governmental terms, with surveillance described as beneficial to the population. Accordingly, inspection practices were constructed as enhancing QI, disciplining and improving the status of the Kenyan paediatric community collectively.

We summarise the four pastoral practices found in CIN in Table 1.

### 5.5 An antidote to frustration with the Kenyan health care context?

Many doctors and nurses we interviewed were motivated by providing good patient care “*The most satisfying thing about my job is when I see my children [patients] going home healthy*” (Paediatrician, 10) and/or developing younger colleagues (“*the satisfying thing has been teaching younger colleagues; to see the transformation from a doctor who had just learnt the theoretical knowledge to actually being able to apply it at the bedside”,* Paediatrician 11).

Yet good intentions were frustrated by the Kenyan health system. Paediatrician 3 noted: *“Frustration is actually top on the list … I wish I had this [equipment/drug], I would be able to save this baby … your potential is being utilized 20–30%, you get bored … your hands are tied.”* This undermined motivation to improve care. Paediatrician 21 noted: *“Motivation … is gone. You go to work at eight and you leave at four. It's just work now, it's not how can I make [health care] better.”*

Despite frustration with the wider Kenyan health care system, interviewees were universally positive about CIN, suggesting that the network provided an oasis of QI and motivation. For example, they described *“a reduction in mortality”* (HRIO 18), *“definite improved quality of care … more staff satisfaction”* (Paediatrician 4), “*motivated people in the hospital”* (Paediatrician 11) and a transformation from not documenting outcomes to documenting “*90–95% of the admissions at discharge”* (Epidemiologist 23). Paediatrician 21 commented on: *“An excellent change for the better in how we are giving care … from the documenting, the reviewing, the adhering to guidelines, the discussions, the sharing of experience, the healthy competition that it has brought.”* Nurse-in-charge 5 described: *“magic within the pediatric department … We are going an extra mile … Having the skills, having the knowledge, it empowers you … with that confidence … you are proud and you want to do it more … it gives you that passion.”*

We reiterate that rather than objectively evaluating CIN, we conducted formative and exploratory research to explain how the network operated. However, in simple evaluative terms (Ferlie et al., 2013), CIN appeared successful in improving clinical outcomes and achieving its stated goals of normalising use of evidence-based guidelines, clinical audit and QI. This was despite operating in a complex and resource-constrained context and CIN playing little role in changing physical structures, providing equipment or financial support for the district hospitals involved (English et al., 2017).

## 6 Discussion and conclusion

We show how the exercise of ‘pastoral power’ and construction of ‘governmentality’ (Foucault, 2007) through complementary ‘pastoral practices’ (Waring and Martin, 2017) facilitated QI within a Kenyan clinical network. While research has used the concept of governmentality to explain clinical networks (Ferlie et al., 2012, Ferlie et al., 2013, Flynn, 2002), we provide a more agentic explanation of how governmentality is constructed by focusing on pastoral practices, while extending Foucauldian analysis of clinical network and QI into LMIC contexts.

First, we describe *constructive practices* developing an evidence-based episteme (based on evidence and evidence-based guidelines) and mechanisms and practices (clinical audit and data collection) making health care provision and outcomes knowable and visible. We suggest that similar constructive practices may be valuable in other LMIC health care systems, where clinical audit and data collection are often ineffective (Kihuba et al., 2014, Tuti et al., 2016). We develop Waring and Martin's (2017) model by showing how network leaders' involvement in constructive practices provides *pastoral status*, which helps leaders to inscribe evidence and audit into practice as we discuss next.

Second, establishing an evidence-based episteme, visibility mechanisms and practices does not ensure their implementation. This relies upon *inscription practices* (Waring and Martin, 2017) that are perceived to help professionals develop individually and collectively and to benefit patients. Elaborating Waring and Martin's model, we describe three novel inscription practices in the network we studied: *championing* and *demonstrating* use of evidence and data collection by evangelizing, teaching and role modelling, and *mentoring/supporting h*ealth professionals.

Third, we describe network leaders facilitating two novel *collective practices*, again elaborating Waring and Martin's (2017) model. First, *meeting and sharing as a professional community* to support and learn from one another. Network members thus created a collective professional community, committed to using evidence, mechanisms and practices making visible how patients are diagnosed, treated and the consequent clinical outcomes. We described a second related collective pastoral practice - *collectively championing and demonstrating evidence-based professionalism*. This led to the development of a new professional social identity, which challenged a traditional authoritarian mode of Kenyan professionalism seen to perpetuate suboptimal professional practices and health care delivery.

Finally, we describe *inspection practices* involving network leaders, local champions and other professionals within the network promoting individual and collective self-discipline to enhance the individual and collective professional status of the Kenyan paediatric community. Where necessary, this constellation of actors disciplined junior colleagues resisting using guidelines or collecting data, checking and testing their use during routine practice until they internalized doing so.

Our findings show the interrelationship between pastoral practices (Waring and Martin, 2017). Constructive practices, creating an evidence and data-based episteme, underpin other pastoral practices. Involvement in constructive practices enhances professionals' pastoral status and inscriptive practices. Professionals then draw upon this episteme during network meetings as a collective practice enhancing professional status and use related inspection practices to discipline themselves and colleagues.

Whereas measurement and transparency are replacing traditional peer-based accountability in health care systems in high income countries (Dixon-Woods et al., 2011, McGivern and Fischer, 2012), this is unlikely in LMICs, due to limited information systems and trust in government authority. Top-down accountability mechanisms may even undermine the ability of those managing local health care systems to respond to patient needs in LMICs (Brown, 2016, Cleary et al., 2013, De Herdt and Oliver de Sardan, 2015, English, 2013). Our findings suggest that *lateral* accountability and governance mechanisms, associated with pastoral practices influencing professional status, may provide a means for motivating health care improvement in LMICs. However, our research is based on a single case study, so further empirical research is needed to comprehensively explain such lateral accountability mechanisms and test the wider generalizability of our tentative hypothesis.

The concept of pastoral practices was developed to explain network leadership (Waring and Martin, 2017). Pastoral practices in CIN involved a constellation of leaders, which can be thought of as a form of *distributed* leadership, found elsewhere to underpin improvement in health care (Denis et al., 2001, Ferlie et al., 2013, Fitzgerald et al., 2013). This pastoral constellation developed through CIN leaders' pastoral practices, which inspired other professionals in district hospitals to become local CIN champions. Some local champions became involved in constructive practices, as part of national guidance panels, but more commonly and significantly, they enacted inscription practices in local district hospital settings, championing, demonstrating, supporting, mentoring and then disciplining evidence and audit-based QI there. Our explanation of how this pastoral constellation of central network leaders and local champions was developed and enacted may explain distributed leadership in other health care settings.

Waring and Latif (2017) similarly describe ‘complementary and competing pastorates’ promoting patient adherence to guidelines for prescribed medicines. They liken doctors to remote ‘shepherds’ supervising and guiding community pharmacists, likened to ‘sheep dogs’, actively ‘herding’ patients (‘sheep’) to become obedient and self-regulating subjects. Similarly, CIN's leaders might be thought of as shepherds guiding local champions herding professionals to internalise evidence and audit based QI techniques in district hospitals. An alternative analogy is network leaders as regional bishops inspiring members of their congregation to become pastors ministering to local communities.

Yet such analogies highlight the fragility of pastoral power, dependent on ‘shepherds’ at the centre of a pastoral constellation. The loss of central network leaders (pastors/shepherds), or their loss of personal or professional credibility and hence pastoral status, may result in local ‘herding’ of clinical practice losing direction or motivation. Supporting junior professionals to develop pastoral status and enact central shepherding roles may mitigate this risk. Yet conflict between competing pastors also presents a risk to pastoral power in terms of loss of direction or motivation. Indeed, while there was little overt conflict in our empirical study, the deferential role of nurse leaders in CIN, vis-a-vis medical leaders, may explain the pockets of resistance to data collection we found.

Finally, returning to the problem we began the paper with, we suggest that the notion of pastoral practices explains social and organizational processes convincing health professionals to implement QI into practice. Yet resources are required to build and preserve activities and relationships to develop and maintain pastoral practices and constellations. This has important implications for the common practice of launching time-limited externally driven programmes without post-programme planning and/or those that fail to engage local institutions so they are prepared and supported to take on roles as shepherds and sheepdogs. Without developing ‘pastoral’ leadership and supporting network activities building and sustaining relationships, pastoral practices and constellations may not emerge, or could collapse prematurely, and the promise of QI techniques to enhance global health may be unfulfilled.

## Financial support

This work was supported by funding from the Wellcome Trust (#097170), who had no role in the empirical research, drafting or submitting this manuscript.
